# A Complex of Badnavirus Species Infecting Cacao Reveals Mixed Infections, Extensive Genomic Variability, and Interspecific Recombination

**DOI:** 10.3390/v12040443

**Published:** 2020-04-14

**Authors:** Roberto Ramos-Sobrinho, Nomatter Chingandu, Osman A. Gutierrez, Jean-Philippe Marelli, Judith K. Brown

**Affiliations:** 1 School of Plant Sciences, The University of Arizona, Tucson, AZ 85721, USA; ramosrs@email.arizona.edu (R.R.-S.); noma.chingandu@wsu.edu (N.C.); 2 USDA-ARS Subtropical Horticulture Research Station, Miami, FL 33158, USA; osman.gutierrez@usda.gov; 3 Mars Wrigley 434 G Street Suite 200, Davis, CA 95616, USA; jean-philippe.marelli@effem.com

**Keywords:** *Badnavirus*, *Caulimoviridae*, dsDNA plant virus, emerging diseases

## Abstract

The incidence of cacao swollen shoot disease (CSSD) in cacao (*Theobroma cacao* L.) has increased in West Africa since ~2000. To investigate the genomic and species diversity of the CSSD-badnaviruses infecting cacao in Côte d’Ivoire and Ghana, symptomatic leaves were subjected to high-throughput sequencing. Among the 30 newly determined genomes, three badnaviruses were identified, *Cacao swollen shoot Togo B virus* (CSSTBV), *Cacao swollen shoot CD virus*, and *Cacao swollen shoot CE virus* (CSSCEV). The phylogenetic trees reconstructed for the reverse transcriptase (RT) and ribonuclease H (RNase H) sequences were incongruent with the complete viral genomes, which had the most robust statistical support. Recombination seems to be involved in the CSSD-badnavirus diversification. The genomic diversity varied among different CSSD-badnaviruses, with CSSTBV showing the lowest nucleotide diversity (π = 0.06236), and CSSCEV exhibiting the greatest variability (π = 0.21911). Evidence of strong purifying selection was found in the coding regions of the CSSTBV isolates.

## 1. Introduction

The Amazon Basin in South America is the center of genetic diversity for cacao (*Theobroma cacao* L.), the source of the cocoa beans. Cacao was introduced to West Africa during the 1880s, and by 1920–1930, had become an economically-viable crop [1,2,3]. Although cacao is grown commercially throughout the tropics, West Africa is the largest supplier of cocoa beans, producing ~70% of the world’s supply [4,5].

Virus-like symptoms were first reported in cacao trees during 1936 in Ghana [6]. Symptoms consisted of discoloration of leaves, vein chlorosis, red vein-banding, stem and root swellings, and pod deformation, with tree decline and death 3–5 years after symptoms development [6,7]. With the expansion of cacao production into other West African countries, outbreaks caused by cacao swollen shoot disease (CSSD) were reported throughout the entire region [8,9,10,11,12,13,14,15,16,17,18]. Disease symptoms reminiscent of CSSD in West Africa were observed in cacao in Sri Lanka in 1956–1957 [19]; however, only recently, the badnavirus *Cacao bacilliform Sri Lanka virus* (CBSLV) was identified in cacao trees showing symptoms similar to those described in the 1950s [15]. Genome sequence comparisons have indicated that the associated virus is highly divergent from CSSD-badnaviruses in West Africa, and represents a unique badnavirus species. Finally, in 1943, virus-like symptoms were observed in commercial cacao fields in Trinidad (Eastern Caribbean). Although foliar discoloration symptoms were prominent in diseased cacao plants from both Trinidad and West Africa, shoot swellings were not observed in Trinidad [20]. Recently, complete genome sequences were determined for two previously uncharacterized badnaviruses, *Cacao mild mosaic virus* (CaMMV) and *Cacao yellow vein banding virus* (CYVBV), which are associated with symptomatic cacao trees in Trinidad [21].

Members of the genus *Badnavirus* (family *Caulimoviridae*) have a circular, double-stranded (ds) DNA genome of 7.0–9.2 kb in length, encapsidated into a non-enveloped bacilliform particle [22]. Badnaviruses replicate through an RNA intermediate using viral-encoded reverse transcriptase [22,23,24], and infect a number of economically important herbaceous and woody crop species. They are transmitted primarily by mealybugs species in a semi-persistent manner [22,25,26,27,28].

The genome of badnaviruses encodes at least three open reading frames (ORFs), referred to as ORFs 1–3 [22,24,29,30,31]. The ORF1 protein is virion-associated [32], and ORF2 of *Cacao swollen shoot Togo B virus* (CSSTBV) encodes a nucleic acid-binding protein [33]. The largest is ORF3, which encodes a polyprotein comprising the viral capsid and movement protein domains, the aspartate protease responsible for polyprotein cleavage, and the reverse transcriptase (RT) and ribonuclease H (RNase H) domains involved in viral replication [24,31]. The cacao-infecting badnaviruses have one to three additional ORFs, known as ORF4, ORFX, and/or ORFY whose functions are still unknown [10,11,14,15,21,30,34].

The genome sequence of CSSTBV was the first CSSD-badnavirus to be determined and characterized [30]. Recently, additional full-length CSSD-badnaviral genomes have been determined from symptomatic cacao plants in West Africa [10,11,14,15,21,34], with ten cacao-infecting badnavirus species being presently recognized by the International Committee on Taxonomy of Viruses (ICTV; https://talk.ictvonline.org/taxonomy/): CBSLV, CaMMV, CYVBV, *Cacao swollen shoot CD virus* (CSSCDV), *Cacao swollen shoot CE virus* (CSSCEV; also known as Cacao red vein virus), *Cacao swollen shoot Ghana M virus* (CSSGMV; also known as Cacao red vein banding virus), *Cacao swollen shoot Ghana N virus* (CSSGNV), *Cacao swollen shoot Ghana Q virus* (CSSGQV), *Cacao swollen shoot Togo A virus* (CSSTAV), and CSSTBV (previously named as Cacao swollen shoot virus).

For decades, strategies designed to combat losses due to swollen shoot disease of cacao have relied on the replacement of infected with uninfected cacao trees. Because the young trees become infected before reaching production age, tree removal programs have not led to economic viability for the long-term, and the practice has provided little to no effect on lowering disease incidence [35,36,37,38]. In previous studies aiming to identify a CSSD resistant cacao germplasm, accessions from the Upper Amazon were reported to exhibit tolerance to two CSSD-inducing badnavirus variants referred to as 1A and New Juaben [39,40]. Recently, SSR analysis has been carried out to identify the genetic background of putative cacao accessions tolerant to CSSD-badnavirus infection in Côte d’Ivoire; however, the identity of the associated badnavirus was not determined [41]. The identification of CSSD tolerant and/or resistant varieties without proper demarcation of the badnavirus species could delay the development of CSSD-resistant germplasms.

Despite the economic importance of CSSD-badnaviruses in West Africa, the causal species and strains and the prevalence and distribution of each are poorly understood. With the increasing number of available cacao-infecting badnavirus genome sequences, species diversity and genomic variability have been shown to be much greater than anticipated. However, this hypothesis is based on a relatively small sample size that is not representative of the entire region [10,11,15,21]. Even so, this type of genomic pattern is consistent with the non-proofreading function associated with the reverse transcriptase encoded by badnaviruses, and their potential for intra- and interspecific recombination [42,43].

To better understand the CSSD epidemiology and provide support for cacao breeding programs involved in the development of resistant varieties for disease management, a regional study is needed to identify and characterize the CSSD-badnavirus complex in West African cacao-growing areas at the complete genome level. An increased understanding of the genetic variability and population-level signatures is essential to better comprehend the badnavirus diversification in relation to disease severity, and to determine the basis for increased CSSD spread beginning in ~2000 [9,14,16,44]. Here, CSSD-badnaviruses associated with symptomatic cacao trees in Côte d’Ivoire and Ghana were characterized using genomic discovery (Illumina) and conventional polymerase chain reaction (PCR) amplification. The resultant 30 new complete genome sequences, together with the 52 cacao-infecting badnaviral genomes available in GenBank, showed genomic signals consistent with diversification involving a range of mutation rates and inter- and intra-species recombination.

## 2. Materials and Methods

### 2.1. Plant Material

Leaf samples of flushing cacao trees exhibiting virus-like symptoms, including foliar discoloration, green and yellow mosaics, vein-clearing, red vein-banding symptoms, and swollen shoot samples, were collected from smallholder farms in five different counties (Kragui, Buyo, Kipiri, Petit Bondoukou, and Petit Bouake) in western Côte d’Ivoire during 2016. Samples from Ghana were collected from smallholder farms where new outbreaks and/or severe symptoms were observed during 2013. Leaves were washed in distilled water, dried, and placed into tubes containing 100% glycerol. The samples were shipped by courier under permit (USDA-APHIS) to The University of Arizona, Tucson, AZ, USA, and stored at 4 °C.

### 2.2. High-Throughput Sequencing and Sequence Assembly

Total DNA was purified from 100 mg of leaf tissue using the method of Doyle and Doyle [45], and subjected to Illumina NextSeq 550 sequencing at the University of Arizona Genetics Core Facility (Tucson, AZ, USA). The NextSeq paired-end libraries (300 bp mean insert size) were constructed using the TruSeq DNA Nano kit (Illumina, San Diego, CA, USA). Reads were demultiplexed and the quality of raw reads was evaluated using FastQC (https://www.bioinformatics.babraham.ac.uk/projects/fastqc/). Removal of adapter sequences was performed using Trimmomatic v.0.32 [46], and a Q score <20 was used to remove low quality bases, with a sliding window size of 4. De novo contig assembly was carried out in SeqMan NGen v.12 (DNASTAR, Madison, WI, USA) with a *kmer* parameter of 21, and up to 2 mismatches. The *T. cacao* nuclear genome sequence contigs were downloaded from GenBank (Accession Nos. CM001879, CM001888, FR722157, and KE132922) [47,48], along with the chloroplast (cacao) and mitochondria (*Gossypium hirsutum* L.) sequences (Accession Nos. HQ244500 and JX065074, respectively), and were used to filter cacao plant sequences, prior to de novo assembly, with a *kmer* of 17, allowing up to 5 mismatches. Also, contigs ≤100 bp were discarded. To additionally evaluate the sequence quality of the assembled contigs, trimmed reads were mapped against the badnavirus-like genome sequences (please see sequence annotation below) using Bowtie2 [49], and manually adjusted in IGV v.2.4.13 [50].

### 2.3. Sequence Annotation

The initial evaluation of the contigs was carried out using the BLASTn algorithm [51] to search the NCBI-GenBank non-redundant nucleotide database for matches to the Illumina-based sequences. Contigs that were about the same size as other well-known badnavirus genomes were selected. The CSSD badnaviral-like genome sequences were manually edited so that the first nucleotide (1) was indicated as the first coordinate of the tRNA^met^ primer binding site. The predicted viral ORFs were located using the ORF Finder algorithm, available at www.ncbi.nlm.nih.gov/orffinder.

### 2.4. Genome Sequence Validation by Sanger DNA Sequencing

To evaluate the accuracy of the de novo-assembled badnavirus genomes, abutting primers were designed based on an apparently full-length genome sequence for one representative isolate of each CSSTBV and CSSCDV isolated from Côte d’Ivoire (Supplementary Table S1). The complete badnavirus genomes were amplified using the CloneAmp HiFi PCR Premix (Clontech, Mountain View, CA, USA) as follows: 1X CloneAmp HiFi PCR Premix, 0.2 μM of each forward and reverse primers, 2 μL of the total DNA (template), and nuclease-free water to a final volume of 25 μL. The cycling conditions were: 2 min at 98 °C as initial denaturation, followed by 35 cycles of denaturation at 98 °C for 20 s, annealing at 55 °C for 15 s, extension at 72 °C for 8 min, with a final extension step at 72 °C for 10 min. The expected size fragments (~7.0 kb) were gel-purified using the illustra GFX PCR DNA and Gel Band Purification kit (GE Healthcare Life Sciences, Uppsala, Sweden) according to the manufacturer’s protocol. The fragments were cloned into the pGEM5 plasmid vector (Promega, Madison, WI, USA), which was previously linearized with the endonuclease *Not*I (New England Biolabs, Ipswich, MA, USA) using the In-Fusion HD Cloning kit (Clontech, Mountain View, CA, USA) and following the manufacturer’s instructions, and it was used to transform *Escherichia coli* DH5α. The plasmid vector was purified using the GeneJET Plasmid Miniprep kit (Thermo Fisher Scientific, Waltham, MA, USA), followed by digestion with *Not*I. Cloned plasmids harboring the expected size insert (~7.0 kb) were sequenced bidirectionally using capillary DNA Sanger sequencing and primer walking with a minimum of 150–200 overlapping bases at Eton Biosciences (San Diego, CA, USA).

Three almost full-length Illumina genomes were found to harbor a truncated ORF3 sequence. To sequence this region, primers were designed to amplify a PCR product spanning the region of interest (Supplementary Table S1). The PCR amplification was carried out using Jumpstart RedTaq ReadyMix (Sigma-Aldrich, Saint Louis, MO, USA), as follows: 1X Jumpstart RedTaq ReadyMix, 0.2 μM of each forward and reverse primers, 2 μL of total DNA (template), and nuclease-free water to a final volume of 25 μL. Cycling conditions were: 2 min at 94 °C for the initial denaturation, followed by 35 cycles of denaturation at 94 °C for 30 s, annealing at 56 °C for 15 s, and extension at 72 °C for 2 min, with a final extension step at 72 °C for 10 min. The expected size fragments were ligated to pGEM-T easy vector (Promega, Madison, WI, USA) and transformed into *E. coli* DH5α. The cloned insert sizes were confirmed by colony PCR using M13 universal primers and were bidirectionally Sanger sequenced by primer walking as previously described. The amplicon and the respective Illumina-contig sequences determined for each isolate were de novo-assembled using Geneious v.8.1.9 (https://www.geneious.com/) prior to further analysis, as described above for Illumina-determined viral genomes.

### 2.5. Badnavirus Species Demarcation

The 52 full-length cacao-infecting badnaviral genomes available in the GenBank database were downloaded (accessed, January 2019; Supplementary Table S2) and aligned with the 30 new complete genomes determined in this study. To confirm taxonomy associated with previously characterized species, and to identify the new isolates, the nucleotide sequences for the complete (1230 bp) RT-RNase H domains were extracted from the complete genomes to create a separate database. Pairwise distance analysis was carried out for the full-length cacao-badnaviral genomes and for the corresponding RT-RNase H sequences, using the Sequence Demarcation Tool v.1.2 software [52]. The ≥80% pairwise nucleotide identity for the RT-RNase H sequences was implemented according to the ICTV-approved threshold for species demarcation within the genus *Badnavirus* [22].

### 2.6. Phylogenetic Inference

Phylogenetic relationships were determined by Bayesian and Maximum Likelihood (ML) analyses by reconstructing phylogenetic trees for the 82 complete viral genomes, and respective RT-RNase H sequences. Historically, the RT-RNase H genomic region has been considered informative at the species level, largely due to its essential role in virus replication, and the ease with which it could be amplified using genus-specific PCR primers together with the lack of complete genome sequence for most badnaviruses [22]. The nucleotide sequence sets were aligned using MUSCLE [53], and edited manually in MEGA7 [54].

The Bayesian phylogenetic analysis was carried out using MrBayes v.3.2 [55] through the CIPRES web portal [56], assuming a general time reversible (GTR) nucleotide substitution model with a gamma (G) model of rate heterogeneity and invariable (I) sites, determined with MrModeltest v.2 [57]. The analysis consisted of two replicates with four chains each for 10 million generations, and sampling every 1000 generations. The first 2500 trees per run were discarded as ‘burn-in’. The posterior probabilities [58] were determined from the majority-rule consensus tree reconstructed with the 15,000 remaining trees. The ML trees were inferred using RAxML v.8 [59] through the CIPRES web portal, assuming a GTR + G evolutionary model. The robustness of individual branches was estimated by 1000 bootstrap replicates. Trees were edited in FigTree v.1.4 (ztree.bio.ed.ac.uk/software/figtree) and Inkscape (https://inkscape.org/pt/). *Grapevine vein clearing virus* (GVCV; Accession No. JF301669) was used as the outgroup.

### 2.7. Recombination Analysis

The possible parental sequences and recombination breakpoints were inferred using the methods RDP, Geneconv, Boot-scan, Maximum Chi Square, Chimaera, SisterScan, and 3Seq implemented in the Recombination Detection Program (RDP) v.4 [60]. The eighty-two aligned complete badnaviral genome sequences were analyzed using default settings for each of the different methods, and statistical significance was inferred by a *p*-value lower than a Bonferroni-corrected cut-off of 0.05. Considering most sequences reported here were Illumina-based, and possible chimeric genomes could interfere in the recombination analysis, an additional data set comprising only complete genomes available from GenBank, and our sequences validated by Sanger sequencing, was analyzed using the RDP4 package as described above. Only recombination events detected by five or more of the different methods were considered to yield a statistically reliable prediction. A second, supporting analysis of the complete genomes that evaluate non-tree-like evolution was carried out using the Neighbor-Net method, implemented in SplitsTree v.4.10 [61].

### 2.8. Genetic Variability and Selection

The mean pairwise number of nucleotide differences per site (diversity, π) was estimated for the complete genomes, and ORFs 1–3, of the CSSTBV (*n* = 40), CSSCDV (*n* = 5), CSSCEV (*n* = 10), and CSSGMV (*n* = 10) badnaviral species using the DNA Sequence Polymorphism (DnaSP) v.6 software [62]. The CSSTBV species was the only one for which a large number of genome sequences (*n* = 40) were available, avoiding the misidentification of selected sites. The amino acid sites evolving under positive or negative selection were analyzed for the ORFs 1–3 of CSSTBV. Two different likelihood methods were used for this analysis: Single-Likelihood Ancestor Counting (SLAC) and Fixed-Effect Likelihood (FEL) [63], implemented in DataMonkey (www.datamonkey.org). To avoid potentially spurious selection estimates possibly due to recombination, the recombination breakpoints were predicted using Genetic Algorithm Recombination Detection (GARD) [64], and the results were used to define the partitions. Bayes factors >50 and *p*-values of <0.1 were considered as the significant cut-off for FEL. The mean ratio of the non-synonymous to synonymous substitutions (*d_N_*/d*_S_*) was calculated using the SLAC method, where *d_N_*/*d_S_* = 1 is indicative of neutral evolution (no selection), *d_N_*/*d_S_* > 1 is indicative of positive selection, and *d_N_*/*d_S_* < 1 is indicating negative selection [65].

## 3. Results

### 3.1. Cacao-Infecting Badnavirus Genomes

Thirty badnavirus full-length genomes were obtained by Illumina DNA sequencing from cacao leaf samples collected in western Côte d’Ivoire (2016, *n* = 117), and Ghana (2013, *n* = 12). The genome sizes ranged from 6978–7314 bp (Table 1). Also, the assembly produced a number of partial badnavirus-like sequences; however, only sequences for which a complete genome was determined were used in the analyses reported here.

The field isolates Buyo17 (CSSTBV) and Buyo2 (CSSCDV) were selected for sequence validation by PCR amplification using isolate-specific primers, cloning, and Sanger DNA sequencing. The two isolates shared 99.9% (Buyo17) and 99.6% (Buyo2) nucleotide (nt) identity with the respective Illumina contig. The complete badnaviral genome sequences exhibited the expected hallmark badnavirus-like features, which include the tRNA^met^ primer binding site, the number and location of predicted ORFs, TATA box, and the polyadenylation-like signal, and together with the >99.5% shared nt identities, showed that the Illumina- and Sanger-determined contigs were in agreement. Sanger sequencing was also used to obtain a specific fragment for three nearly full-length Illumina-genomes that harbored a truncated ORF3: CSSCDV (Kipi7a (1841 bp), and Kipi10a (1805 bp)) and CSSCEV (Ghana1 (2033 bp)). After assembling the Illumina and Sanger partial sequences, the isolates Kipi7a, Kipi10a, and Ghana1 had an intact, in-frame ORF3. Based on an alignment of the full-length sequences, and identification of coding regions, the genome organization of the 30 new complete genomes were found to be characteristic of the respective previously characterized CSSD-badnaviral species. The CSSTBV isolates encoded 5–6 predicted ORFs (ORFs 1–4, X and Y), CSSCEV isolates showed 4 ORFs (ORFs 1–3 and Y), while CSSCDV isolates had 5 predicted ORFs (ORFs 1–3, X, and Y).

### 3.2. Species Diversity of Cacao-Infecting Badnaviruses

Taxonomically, the RT-RNase H genomic region has been considered informative at the species level, and has been approved by the ICTV for species demarcation in the genus *Badnavirus* [22]. Besides the approximate size of 1230 bp of the RT-RNase H domains, some studies have reported pairwise nucleotide comparisons for partial sequences (~580 bp). Here, only the complete fragment of the RT-RNase H was analyzed. The pairwise distances for the RT-RNase H domains indicated that the isolates shared 61.5 to 81.1% nt identity. In comparison, the full-length genome sequences shared 59.0 to 79.7% nt identity (Supplemental Table S3). Based on the genome sequence analysis, the species groups were largely consistent with those defined based on the RT-RNase H. The 30 newly determined genomes were assigned to three previously described badnaviral species: CSSTBV (*n* = 25, Côte d’Ivoire), CSSCDV (*n* = 3, Côte d’Ivoire), and CSSCEV (*n* = 2, Ghana). Two cacao samples (Kipi7 and Kipi10) were found to harbor a mixed infection of CSSTBV (isolates Kipi7b and Kipi10b) and CSSCDV (isolates Kipi7a and Kipi10a) (Table 1).

The 52 genomes available in GenBank were clustered into groups containing seven recognized West African CSSD species (CSSCDV, CSSCEV, CSSGMV, CSSGNV, CSSGQV, CSSTAV, and CSSTBV) [10,11,14,15,30], the two cacao-infecting badnaviruses from Trinidad (CaMMV and CYVBV) [21], and CBSLV from Sri Lanka [15]. Among the CSSD-badnavirus species for which genome sequences were available, CSSTBV represented the largest number (*n* = 40 isolates), which was considered to comprise a population.

### 3.3. Phylogenetic Relationships

**Figure 1:**
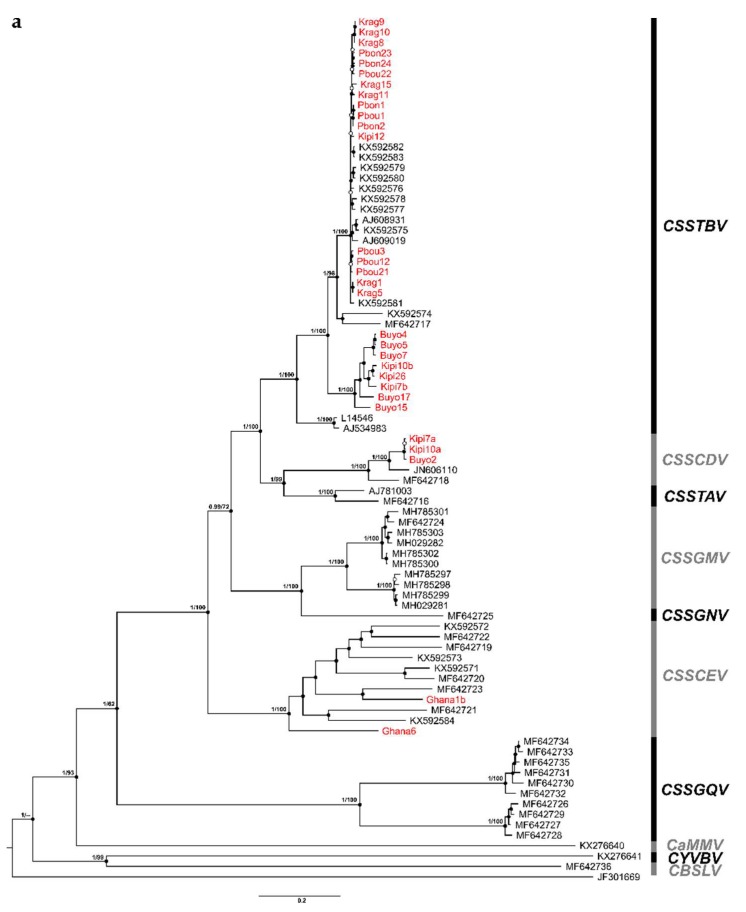

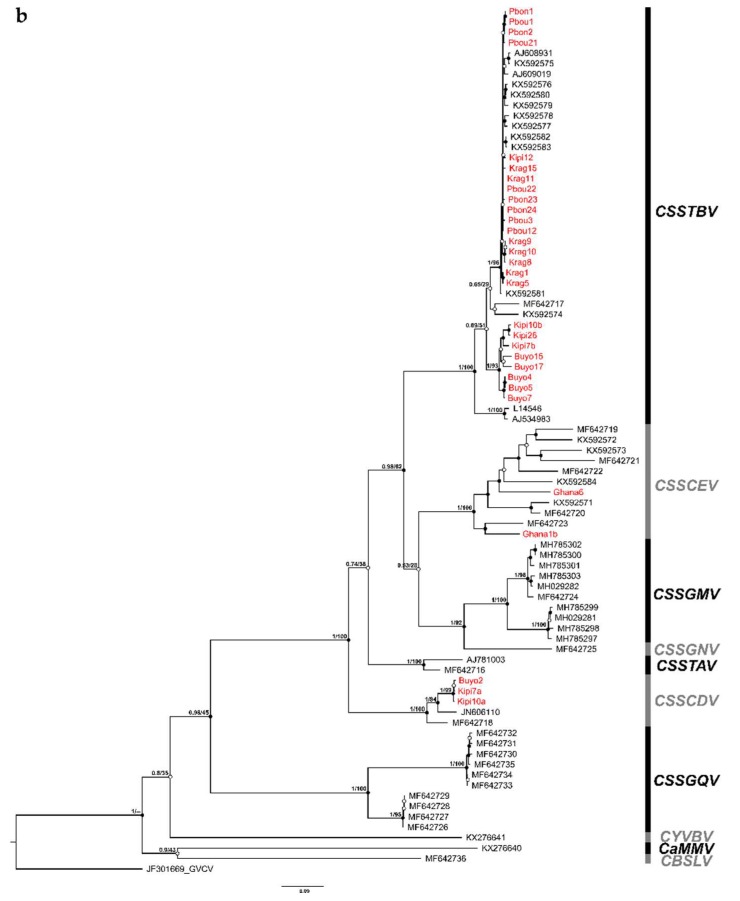
Bayesian phylogenetic trees based on complete genome (**a**) and reverse transcriptase and ribonuclease H (**b**) nucleotide sequences of badnaviruses infecting cacao. Posterior probability (left) and bootstrap (right) support values are shown above the main tree nodes. Posterior probabilities are also represented by filled (0.95–1.00) and empty (0.50–0.94) circles. Sequences reported in this study are highlighted in red. *Cacao bacilliform Sri Lanka virus* (CBSLV), *Cacao mild mosaic virus* (CaMMV), *Cacao yellow vein banding virus* (CYVBV), *Cacao swollen shoot CD virus* (CSSCDV), *Cacao swollen shoot CE virus* (CSSCEV), *Cacao swollen shoot Ghana M virus* (CSSGMV), *Cacao swollen shoot Ghana N virus* (CSSGNV), *Cacao swollen shoot Ghana Q virus* (CSSGQV), *Cacao swollen shoot Togo A virus* (CSSTAV), and *Cacao swollen shoot Togo B virus* (CSSTBV). *Grapevine vein-clearing virus* (GVCV) was used as an outgroup.

The Bayesian and ML phylogenetic trees based on full-length genomes were in concordance with one another, and agree with previous reports showing that most CSSD-badnaviruses from West Africa are more closely related to each other than to other badnaviruses known to infect cacao elsewhere, with the exception of the CSSGQV isolates. The main clades resolved by the Bayesian phylogenetic tree for the known cacao-infecting badnavirus species were well-supported by posterior probabilities (pp) of 0.99–1.00 (Figure 1a), and bootstrap values were >70% for all of the ML tree clades, except the clade containing the CSSGQV isolates, which was separate from all the other badnaviruses in West Africa, with a 62% bootstrap value (Figure 1a). This distinct clade either represents a divergent West African lineage in relation to others previously known from the region or represents an introduction. Nonetheless, reconstructed complete genome phylogeny for cacao-infecting badnaviruses showed robust statistical support. The 30 newly reported isolates (this study) were clustered in three different groups, with 25 isolates grouping in the CSSTBV clade, three isolates in the CSSCDV clade, and two of the isolates clustered in the CSSCEV clade. The CSSGMV isolates formed a sister group to CSSGNV. The CYVBV and CBSLV isolates grouped separately from the West African clades, to which they were basal (Figure 1a).

The Bayesian and ML phylogenetic trees for the complete RT-RNase H resolved all of the same monophyletic species clades observed in the complete genome trees, with high pp (1.00) and robust bootstrap (100%) values. However, the tree topology predicted for the RT-RNase H sequences was incongruent with the complete genome, also having lower statistical support (Figure 1b). The CSSCEV isolates were positioned as a sister clade to CSSGMV and CSSGNV in the RT-RNase H tree, which had very low pp (0.63) and less than acceptable bootstrap support at 28%. Also, CSSCDV and CSSTAV were resolved as distinct clades, and had a low pp of 0.74, and bootstrap values were only 38% for the CSSTAV clade (Figure 1b).

### 3.4. Recombination Affecting the Diversification of Cacao-Badnaviruses

**Figure 2:**
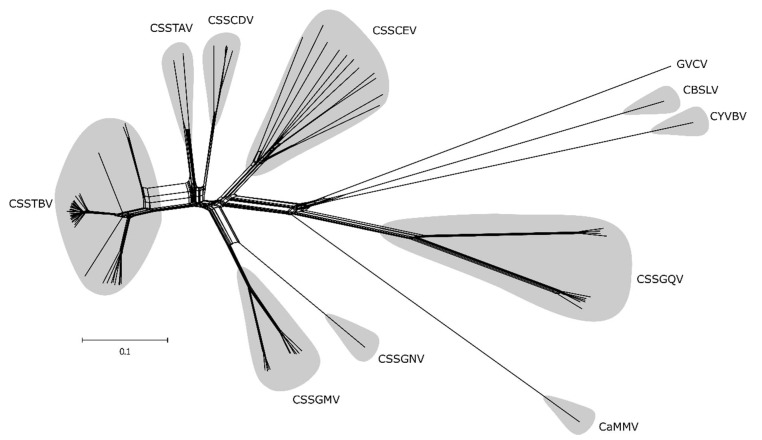
Phylogenetic network based on complete genome nucleotide sequences of cacao-infecting badnaviruses, constructed using the Neighbor-Net algorithm. Reticulation among the viral species are shown by parallel paths instead of a bifurcating evolutionary tree, indicating putative recombination. Cacao-infecting badnaviral species are highlighted in grey. *Cacao bacilliform Sri Lanka virus* (CBSLV), *Cacao mild mosaic virus* (CaMMV), *Cacao yellow vein banding virus* (CYVBV), *Cacao swollen shoot CD virus* (CSSCDV), *Cacao swollen shoot CE virus* (CSSCEV), *Cacao swollen shoot Ghana M virus* (CSSGMV), *Cacao swollen shoot Ghana N virus* (CSSGNV), *Cacao swollen shoot Ghana Q virus* (CSSGQV), *Cacao swollen shoot Togo A virus* (CSSTAV), *Cacao swollen shoot Togo B virus* (CSSTBV), and *Grapevine vein-clearing virus* (GVCV).

The putative effect of recombination among the complete genomes of cacao-infecting badnaviruses was evaluated by the neighbor-net approach. The phylogenetic networks indicated conflicting signals, which are represented by the several boxes/parallel paths, characteristically resulting from prior recombination events. The reticulation was most pronounced among the three West African species CSSTBV, CSSTAV, and CSSCDV (Figure 2). Also, by this analysis, long branch lengths were observed for several species, and were most evident among the CSSCEV and non-West African species (Figure 2), which can be caused by accumulating mutations. In addition, the analysis showed a closer genetic relationship among CSSGQV isolates and the non-West African species than with the other groups (Figure 2).

To identify the putative recombination breakpoints and prospective parental sequences, the 82 available cacao-infecting badnavirus genomes were analyzed using the RDP4 package. Based on stringent criteria, at least eight independent recombination events were predicted among badnaviral species from West Africa. Recombination was detected among the species CSSTBV, CSSCDV, and CSSTAV. Many of the recombination breakpoints were located between the intergenic region (IR) and ORF2, with CSSTBV isolates identified as the putative recombinant sequences (events 1, 2, 3, 5, and 7) (Supplementary Table S4). For the isolates Kipi7 and Kipi10, harboring a mixed infection consisting of CSSTBV and CSSCDV, the isolates Kipi7b and Kipi10b (CSSTBV) were identified as putative recombinants that had Kipi7a (CSSCDV) as an inferred minor parent, with recombination breakpoints located in the IR and ORF1 regions of the genome (event 2) (Supplementary Table S4). A recombination event (6) with breakpoints located at the nucleotide coordinates 4980 and 6340 (ORF3) was present in all of the CSSTBV genomes analyzed here, which is indicative of a lineage-specific event, with a CSSCEV-like ancestor inferred as the minor parent (Supplementary Table S4). Intraspecies recombination was detected in the CSSCEV (Accession No. KX592573) isolate, and the recombination breakpoints were located at the nucleotide coordinates 3925 and 5053 (ORF3) of the genome (event 8; Supplementary Table S4). The CSSGQV isolate (Accession No. MF642731) was identified as a parent involved in intraspecies recombination, with breakpoints located at nucleotide coordinates, 6959 and 229, located within the IR (event 4; Supplementary Table S4).

It can be observed that most recombination events were detected among sequences previously reported and available in GenBank, which were also validated by Sanger sequencing (events 1, 5, 7, and 8), and that the recombination event 6 was detected not only in the Illumina-based sequences reported here, but it was also shared by CSSTBV genomes from GenBank. Finally, the two unique recombination events occurring only among the new genomes reported here (events 2 and 3) were also present in the Sanger-sequenced genome (CSSTBV, isolate Buyo17), reinforcing that these recombination events may be real. To additionally support the recombination pattern detected among cacao-infecting badnaviruses, a data set comprising only complete genomes available from GenBank, and our sequences validated by Sanger sequencing, was also analyzed. At least six independent recombination events were predicted among CSSD-badnaviruses from West Africa. Similarly, a complex pattern of predicted recombination events was observed, mainly involving CSSTBV, CSSTAV, and CSSCDV isolates. A recombination event (1) with breakpoints located at the nucleotide coordinates 93 (IR) and 852 (ORF2) was predicted in the isolate Buyo17 (CSSTBV), which resembles the recombination events 2 and 3 reported above (Supplementary Tables S4 and S5). CSSTBV isolates were identified as the putative recombinant sequences of two independent recombination events (2 and 4), with predicted breakpoints located between the IR and ORF2 (Supplementary Table S5). Also, two recombination events with breakpoints located at the nucleotide coordinates 2265 and 3687 (ORF3; event 3) and 4473 and 6460 (ORF3; event 5) were present in CSSGQV and CSSCDV isolates, respectively.

### 3.5. Genetic Variability in CSSD-Badnavirus Populations

For genetic variability analysis, the four best-known badnaviral species were considered as the following distinct subpopulations: CSSTBV (*n* = 40), CSSCEV (*n* = 10), CSSGMV (*n* = 10), and CSSCDV (*n* = 5). The per-site nucleotide diversity (π) estimate was lowest for CSSTBV isolates (π = 0.06236), while CSSCDV and CSSGMV showed moderate diversity at π = 0.07111 and π = 0.08393, respectively, and CSSCEV (π = 0.21911) exhibited the greatest nucleotide variability (Table 2). The variability was unevenly distributed across the genomes, with ORF2 showing higher values than for ORFs 1 and 3, separately, or compared to complete genome sequences (Table 2). For CSSTBV in particular, the highest nucleotide diversity values were observed in ORFs 1 and 2 at π = 0.08312 and π = 0.09505, respectively, compared to ORF3 with π = 0.06245 (Table 2).

### 3.6. Purifying Selection Acting on CSSTBV Population

**Figure 3:**
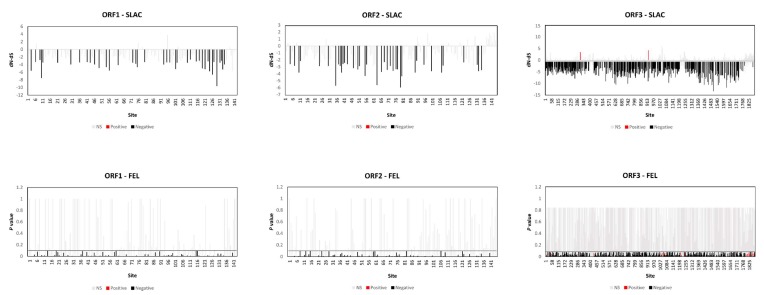
Positively and negatively selected sites in the open reading frames (ORFs) 1–3 of the *Cacao swollen shoot Togo B virus* (CSSTBV) population. *d_N_*-*d_S_* > 0 indicates positive selection, while *d_N_*-*d_S_* < 0 suggests negative selection for Single-Likelihood Ancestor Counting (SLAC). Sites with *p*-value < 0.1 were considered statistically significant for Fixed-Effect Likelihood (FEL). Sites under statistically detectable positive or negative selection are highlighted in red and black, respectively, and not significant is in grey.

The effect of selection pressure on genetic variability among the CSSTBV isolates was evaluated by the SLAC and FEL methods [62]. All of the coding regions of CSSTBV showed low *d_N_*/*d_S_* mean ratios, *d_N_*/d*_S(ORF1)_* = 0.102, *d_N_*/*d_S(ORF2)_* = 0.204, and *d_N_*/*d_S(ORF3)_* = 0.134, indicative of a strong purifying selection. For ORF1, the SLAC (39 sites) and FEL (66 sites) methods detected only sites under statistically significant negative selection, and sites detected by SLAC were also identified by FEL. The results for ORF2 were similar in that SLAC and FEL detected 38 and 64 negatively selected sites, respectively. Also, a large number of sites under negative selection were identified in ORF3 by both SLAC (459 sites) and FEL (804 sites). The SLAC method detected positive selection in ORF3 at two sites, compared to 24 sites detected by FEL. These results indicate that negative selection was the most important selective force shaping genetic variability in CSSTBV isolates (Figure 3).

## 4. Discussion

Viruses belonging to the genus *Badnavirus* infect economically important crops worldwide. In West Africa, CSSD-badnaviruses have been reported to reduce the quality of cacao beans and overall yield [35,38,66,67]. Despite nearly 90 years of research centered on the swollen shoot disease of cacao in West Africa, only recently a number of divergent CSSD-associated species and strains have been identified. Even so, additional information is needed with respect to genomic variability to enable the development of reliable molecular detection assays for breeding programs and epidemiologically-based management [10,11,14,15,21,30,39,68,69,70]. Also, population-level analysis has been limited to a relatively few complete genomes and/or partial sequences [9,14,16,34,66]. In this study, 30 new CSSD-badnavirus genome sequences were determined from symptomatic cacao trees in Côte d’Ivoire and Ghana using Illumina and/or Sanger DNA sequencing. High genomic diversity was found among CSSD-badnavirus subpopulations, as well as evidence for intra- and inter-specific recombination.

Pairwise distances of the complete RT-RNase H sequences (1230 bp) for 82 cacao-infecting badnaviral genomes, and the ICTV-approved ≥80% nt identity cut-off, identified 10 distinct species. Among the 30 newly determined CSSD-genomes, CSSTBV (*n* = 25) was the predominant species in Côte d’Ivoire, while CSSCDV was the second most abundant (*n* = 3). The recently described species, CSSCEV, was the only species identified infecting samples from Ghana (*n* = 2). Together with all of the available complete genome sequences determined recently from West Africa [10,11,14,15,30,34], CSSTBV was the prevalent species infecting cacao. Although CSSTBV was represented by the greatest extent among the samples analyzed from West Africa, a complex of badnaviral species is known to be involved with CSSD. Thus, efforts to mitigate losses caused by this economically important viral group should consider the extant genetic diversity among the known viruses, and should carry out additional studies to characterize the complete species complex of the causal agent.

The phylogenetic tree topologies reconstructed for the CSSD-badnaviral RT-RNase H and full-length genome sequences were shown to be incongruent, and further, the RT-RNase H trees lack robust statistical support (0.52–1.00 pp and 28–100% bootstrap values) to resolve the phylogenetic clustering of all so far known cacao-infecting badnavirus species as observed for the more informative complete genomes (0.99–1.00 pp and 62–100% bootstrap values). The monophyletic viral species clades were well-resolved, regardless of whether the analyses considered the complete genome or RT-RNase H regions, based on 1.00 pp and bootstrap values of 91–100%. The results indicate that although the reconstruction of the phylogenetic relationship among cacao-infecting badnaviruses based on the RT-RNase H nt sequences is less robust when compared to the complete viral genome, pairwise nt sequence comparisons of the RT-RNase H region, and the ≥80% identity cutoff, were suitable for species demarcation of the cacao badnaviruses. However, this criterion may be of limited value for distinguishing species when recombination events occur within the RT-RNase H. Due to increasing availability of badnaviral genome sequences in GenBank, it would seem prudent to revisit the criteria for species demarcation for the genus *Badnavirus*, such that information for the genus is updated regularly, as has been accomplished for other plant virus groups.

The establishment of mixed viral infections in plant hosts is a requisite for recombination among different species or strains [71,72], and cacao trees have been shown to harbor mixed infections of CSSD-badnaviruses [10,15]. In this study, at least eight independent recombination events were identified among CSSD-badnaviruses, particularly, with CSSTBV as the recombinant recipient and CSSCDV or CSSTAV as inferred parents, and with most recombination breakpoints occurring between the IR and ORF2. Additional, unique intraspecies recombination events were detected in the CSSGQV and CSSCEV isolates, also having breakpoints in the IR and ORF3, respectively. Recombination has been reported among yam-, banana- and sugarcane-infecting badnaviruses, with similar genomic locations of recombination breakpoints [73,74]. Together, the results demonstrate that recombination is an important evolutionary mechanism among cacao-infecting badnaviruses, with hotspots of recombination occurring in the IR and ORF2/C-terminal of ORF3. Also, some of the CSSTBV and CSSCDV sequences identified as recombinants and/or as sequence donors/parents were recovered from double-infected cacao plants, providing evidence that recombination is occurring among extant cacao-infecting badnaviruses. Although it is well-known that wild plant species have served as the initial, primary sources of CSSD-badnaviruses in West Africa [9,28,75], representative badnaviral genome sequences from them are needed to pursue evolutionary and epidemiological questions and the patterns of diversification among isolates extant in wild host species. It is also important to understand which (if any) of them may continue to serve as CSSD-badnavirus reservoirs throughout the region.

Caulimoviruses accumulate nucleotide substitutions similar to the rates associated with RNA and single-stranded (ss) DNA viruses, a phenomenon that may be due to the lack of the inherent proofreading activity of the reverse transcriptase enzyme [43,73,76]. Together with mutation, recombination influences the extents of virus variability observed across and between populations [42,73].

In this study, high nucleotide diversity, which is comparable to the genetic variability reported for the banana- and sugarcane-infecting badnaviruses [73], was observed among the CSSD-badnaviruses, corroborating the previous reports of high molecular variability based on analysis of partial CSSD-like genome sequences [16,34]. Also, high diversity was associated with the ORF2 of CSSD-badnavirus populations compared to ORFs 1 and 3 diversity estimates. The ORFs 1 and 2 were the most variable genomic regions among the CSSTBV isolates, which is consistent with evidence provided here that these genomic regions were hotspots of recombination. The extensive genomic diversity has complicated the design of molecular diagnostic assays that are able to detect and/or identify the suspected extensive range of causal virus species/strains. Thus, the inability to provide confirmation of badnavirus presence and causality, whether symptoms are observed or not, has limited crucial epidemiological assessments, and therefore, has hindered knowledge-based disease management.

Isolates of the CSSCEV species showed the most extensive genomic variability among all of the CSSD-badnavirus populations studied so far, which is most probably due to high rates of mutation and/or recombination. It has been suggested that CSSCEV has emerged only recently in cacao (~1990), perhaps representing the most recent host-shift occurring in cacao from the wild/primary reservoir, a conclusion based on the extreme severity of symptoms, which would have not gone unnoticed if the rapid decline isolates had emerged previously. Rapid tree decline, e.g., within as little as one-year post-symptom development, has been associated with CSSCEV sequences from trees along the border of western Ghana and eastern Côte d’Ivoire, which is suggestive of the zone of emergence [10]. A host jump, and a recent population expansion in cacao, could readily explain the high nucleotide diversity observed among CSSCEV isolates. To test this hypothesis, additional CSSCEV genome sequences are needed from cacao plants and suspected, putative wild hosts associated with cacao farms. Further, CSSCEV isolates have been implicated in inter- and intra-species recombination, which is strongly suggestive that recombination was an important evolutionary mechanism influencing the extant genetic variability of CSSCEV populations. Presently, it is not possible to determine whether this extreme state of genomic variability observed for CSSCEV subpopulations has achieved an ‘equilibrium of variability’ that might be expected to persist over the next decades, especially if the CSSCEV subpopulation has experienced a recent expansion in cacao. This information is important considering the extent of durability of cacao genotypes and clones planted in commercial production fields, which are expected to produce cacao pods for the long term.

The badnaviral ORFs 1–2 products have been reported to function as virion-associated/nucleic acid-binding proteins; however, their biological function(s) in viral life cycle is still unknown [32,33]. Despite the observed high nucleotide diversity in ORFs 1–2 of CSSTBV isolates, strong negative selection appears to have preserved the amino acid sequences of the viral proteins. Purifying selection was also identified as the primary force of selection associated with ORF3 of CSSTBV isolates, based on evidence of infrequently occurring sites under positive selection. ORF3 encodes a polyprotein with domains for viral RT-RNase H, aspartate protease, and capsid and movement proteins [24,31]. Conservation of the latter protein domains may be crucial for vector-mediated transmission (capsid protein) and systemic infection (movement protein). By comparison, negative selection also has been implicated as the main selective force acting on banana- and sugarcane-infecting badnavirus populations [73]. Taken together, the results illustrate that the high nucleotide diversity in these viral groups is subject to strong purifying selection to retain the vital functions of the badnaviral proteins.

The widespread distribution of phylogenetically related CSSTBV isolates throughout distantly associated geographic regions in West Africa reinforces the hypothesis that CSSTBV is of West African endemism and may infect some of the same plant hosts that occur throughout the region. Cacao was introduced to West Africa during the 1880s, and following the adaptation of CSSTBV to cacao, the virus spread in cacao mediated by mealybug vector transmission and/or by CSSTBV-infected germplasm transported into the cacao-growing region. The exchange of cacao germplasm between and within the different growing areas could have occurred frequently or less-so, nonetheless contributing to the regional distribution/dispersal of CSSTBV across much of the region. Presently, the majority of available complete genome sequences represent isolates collected in Côte d’Ivoire. This underscores the need for additional and regionally-representative sampling of both CSSTBV isolates to ascertain endemism, and of all other CSSD-species extant in all cacao-growing areas of West Africa, to elucidate the patterns and extent of geographical structuring of badnaviral populations associated with cacao and uncultivated CSSD-badnaviral host species.

## 5. Conclusions

Despite the ICTV-approved use of the RT-RNase H as the sequence of choice for badnaviral species demarcation, the low statistical posterior probability and bootstrap support values for the RT-RNase H Bayesian and ML trees, respectively, showed that the RT-RNase H region harbors a phylogenetic signal insufficient in reflecting the evolutionary relationships of cacao-infecting badnaviruses, and it conveys a poorly resolved phylogeny that is strikingly different from the well-supported phylogenies reconstructed for the corresponding complete genome sequences. Among the CSSD-subpopulations examined here, CSSCEV was the most variable, and this is probably attributable to its recent emergence, and a hypothesis that its genomic signals reflect high mutation rates and recombination. Similarly, high levels of nucleotide diversity in CSSTBV and CSSCDV are apparently related to a propensity for intra- and inter-species recombination. With the discovery that the accumulation of mutations and evidence of recombination have given rise to extensive genomic variability among CSSD-badnaviruses in West Africa, it may be predicted that new and more aggressive variants will emerge in cacao with great potential to undermine cacao production, while at the same time complicating disease management for this export perennial crop that provides an important lifeline to most of West Africa.

## Figures and Tables

**Table 1:** Cacao swollen shoot disease (CSSD)-badnavirus isolates obtained from cacao leaf samples collected in Côte d’Ivoire and Ghana between 2013 and 2016.

Isolate	Viral Species (Acronym)	GenBank Accession #	Genome Size (bp)	# of Mapped Reads	Depth of Coverage	Location	Date
Buyo4	*Cacao swollen shoot TB virus* (CSSTBV)	MN433941	7045	10,988	224	Côte d’Ivoire	2016
Buyo5	CSSTBV	MN433942	7045	8680	173	Côte d’Ivoire	2016
Buyo7	CSSTBV	MN433940	7055	19,224	407	Côte d’Ivoire	2016
Buyo15	CSSTBV	MN433938	7090	4572	93	Côte d’Ivoire	2016
Buyo17 *	CSSTBV	MN433939	7066	18,809	398	Côte d’Ivoire	2016
Kipi7b	CSSTBV	MN433943	7080	5850	110	Côte d’Ivoire	2016
Kipi10b	CSSTBV	MN433944	7055	4641	94	Côte d’Ivoire	2016
Kipi12	CSSTBV	MN433947	7007	10,509	225	Côte d’Ivoire	2016
Kipi26	CSSTBV	MN433945	7064	16,526	315	Côte d’Ivoire	2016
Krag1	CSSTBV	MN433948	7027	944	21	Côte d’Ivoire	2016
Krag5	CSSTBV	MN433949	7027	3872	83	Côte d’Ivoire	2016
Krag8	CSSTBV	MN433953	7023	7893	159	Côte d’Ivoire	2016
Krag9	CSSTBV	MN433954	7041	2954	61	Côte d’Ivoire	2016
Krag10	CSSTBV	MN433955	7023	9397	188	Côte d’Ivoire	2016
Krag11	CSSTBV	MN433959	7022	6174	132	Côte d’Ivoire	2016
Krag15	CSSTBV	MN433946	7063	636	14	Côte d’Ivoire	2016
Pbon1	CSSTBV	MN433961	7024	7408	157	Côte d’Ivoire	2016
Pbon2	CSSTBV	MN433960	7018	5274	103	Côte d’Ivoire	2016
Pbon23	CSSTBV	MN433957	7030	7793	166	Côte d’Ivoire	2016
Pbon24	CSSTBV	MN433958	7035	90,991	1917	Côte d’Ivoire	2016
Pbou1	CSSTBV	MN433962	7072	11,749	230	Côte d’Ivoire	2016
Pbou3	CSSTBV	MN433951	7024	10,489	225	Côte d’Ivoire	2016
Pbou12	CSSTBV	MN433952	7018	16,193	340	Côte d’Ivoire	2016
Pbou21	CSSTBV	MN433950	7031	7815	167	Côte d’Ivoire	2016
Pbou22	CSSTBV	MN433956	7040	9496	180	Côte d’Ivoire	2016
Buyo2 *	*Cacao swollen shoot CD virus* (CSSCDV)	MN433935	7211	53,531	1093	Côte d’Ivoire	2016
Kipi7a †	CSSCDV	MN433936	7212	17,943	338	Côte d’Ivoire	2016
Kipi10a †	CSSCDV	MN433937	7173	6622	132	Côte d’Ivoire	2016
Ghana6	*Cacao swollen shoot CE virus* (CSSCEV)	MN433933	7314	14,366	278	Ghana	2013
Ghana1b †	CSSCEV	MN433934	6978	2151	44	Ghana	2013

* Complete genomes were validated by Sanger sequencing. † Partial genome sequences were obtained by Sanger sequencing.

**Table 2:** Nucleotide diversity of the complete genome of badnavirus isolates/species infecting cacao.

Species ҂	No. of Sequences	Haplotypes	Nucleotide Diversity *	SD †
CSSTBV_(genome)_	40	40	0.06236	0.00948
CSSTBV_(ORF1)_	40	40	0.08312	0.01329
CSSTBV_(ORF2)_	40	40	0.09505	0.01354
CSSTBV_(ORF3)_	40	40	0.06245	0.00936
CSSCEV_(genome)_	10	10	0.21911	0.01159
CSSCEV_(ORF1)_	10	10	0.21785	0.01254
CSSCEV_(ORF2)_	10	10	0.24865	0.01474
CSSCEV_(ORF3)_	10	10	0.22018	0.01209
CSSGMV_(genome)_	10	10	0.08393	0.01108
CSSGMV_(ORF1)_	10	10	0.07212	0.01011
CSSGMV_(ORF2)_	10	10	0.10199	0.01412
CSSGMV_(ORF3)_	10	10	0.08498	0.01102
CSSCDV_(genome)_	5	5	0.07111	0.02316
CSSCDV_(ORF1)_	5	5	0.06227	0.01832
CSSCDV_(ORF2)_	5	5	0.09065	0.03089
CSSCDV_(ORF3)_	5	5	0.07097	0.02362

҂ *Cacao swollen shoot Togo B virus* (CSSTBV), *Cacao swollen shoot CE virus* (CSSCEV), *Cacao swollen shoot Ghana M virus* (CSSGMV), and *Cacao swollen shoot CD virus* (CSSCDV). * Pairwise, per-site nucleotide diversity. † Standard deviation.

## Supplementary Materials

Supplementary materials can be found at https://www.mdpi.com/1999-4915/12/4/443/s1.
