# Supplementary material for: A Complex of Badnavirus Species Infecting Cacao Reveals Mixed Infections, Extensive Genomic Variability, and Interspecific Recombination
Source: Viruses. 2020 Apr 14;12(4):443. doi: 10.3390/v12040443 (PMC7232428; doi:10.3390/v12040443)
Supplement: Supplementary file 1 [file viruses-12-00443-s001.pdf]

Supplementary Material

Supplementary Table S1. Primer pairs used for amplification of complete and partial genomes of cacao-infecting badnaviruses in West Africa.

Primers	Sequence	Target	Tm (°C)	Amplicon
Abutting Primers				
CSSTBV-Buyo17-F	GAATTCATAGCTGTGTACATTGATG	CSSTBV	55	7.0kb
CSSTBV-Buyo17-R	GAATTCTTCTGTGCCTTTGAAAC			
CSSCDV-Buyo2-F	GTCGACAGGACGTTTATACAACC	CSSCDV	55	7.2kb
CSSCDV-Buyo2-R	GTCGACTTGATTTCCTCCGAC			
Partial sequences				
CSSCEV-Ghana1b-F	GTTCTGAGACCACCACAGGCAG	CSSCEV	56	2.0kb
CSSCEV-Ghana1b-R	GGCTTTGGCTGGACTGTTATATCAC			
CSSCDV-Kipi7a/10a-F	GAAGTAGCCATCCCAGAAGAC	CSSCDV	56	1.8kb
CSSCDV-Kipi7a/10a-R	CACTTGGTATCCTTCCTTCGTATAG			
CSSTBV-CIS3-F	GGCACAATCATCTCTCAAGGTC	CSSTBV	56	0.8kb
CSSTBV-CIS3-R	CGTGAGTAATGGCGTCCTTC			

Supplementary Table S2. Complete genome sequences of cacao-infecting badnaviruses retrieved from GenBank (accessed on January 2019).

Species	Acronym	GenBank accession Nos.
Cacao swollen shoot Togo B virus	CSSTBV	L14546, AJ534983, MF642717, KX592574, AJ608931, KX592575, AJ609019, KX592578, KX592577, KX592576, KX592579, KX592580, KX592581, KX592582, KX592583
Cacao swollen shoot CD virus	CSSCDV	MF642718, JN606110
Cacao swollen shoot Togo A virus	CSSTAV	AJ781003, MF642716
Cacao swollen shoot CE virus	CSSCEV	MF642723, MF642719, MF642722, KX592572, KX592571, MF642720, MF642721, KX592584, KX592573
Cacao swollen shoot Ghana M virus	CSSGMV	MH785299, MH029281, MH785298, MH785297, MH785301, MF642724, MH785303, MH029282, MH785302, MH785300
Cacao swollen shoot Ghana N virus	CSSGNV	MF642725
Cacao swollen shoot Ghana Q virus	CSSGQV	MF642726, MF642727, MF642729, MF642728 MF642730, MF642735, MF642734, MF642733, MF642732, MF642731
Cacao mild mosaic virus	CaMMV	KX276640
Cacao yellow vein banding virus	CYVBV	KX276641
Cacao bacilliform Sri Lanka virus	CBSLV	MF642736

Supplementary Table S3. Pairwise nucleotide sequence identities among cacao-infecting badnaviruses based on complete (1230 bp) RT-RNase H sequences, and full-length genomes, using the Sequence Demarcation Tool (SDT) program. The range of percent nucleotide identities are shown.

RT-RNase H complete (1230 bp)													
	**CSSGQV**	**CSSGRV***	**CRVV†**	**CSSCEV**	**CSSCDV**	**CSSTAV**	**CSSTBV**	**CRVBV⁑**	**CSSGMV**	**CSSGNV**	**CaMMV**	**CYVBV**	**CBSLV**
**CSSGQV**	99.7-99.9												
**CSSGRV***	80.5-81.1	98.9-100											
**CRVV†**	64.4-66.3	64.0-66.9	82.1-85.5										
**CSSCEV**	64.6-67.6	64.3-65.7	80.9-94.7	81.8-86.8									
**CSSCDV**	66.0-68.0	64.5-66.5	71.8-75.0	71.5-74.0	90.7-99.6								
**CSSTAV**	65.6-68.4	65.5-66.8	74.8-77.4	74.0-76.3	75.9-77.8	90.1							
**CSSTBV**	63.8-66.5	63.1-66.1	74.3-77.9	74.5-78.8	73.5-75.8	74.5-77.5	87.6-100						
**CRVBV⁑**	66.4-67.5	64.1-66.3	72.3-76.9	72.3-76.3	72.8-75.1	73.8-76.5	73.6-76.6	87.3-99.9					
**CSSGMV**	67.1-67.3	65.2-65.6	74.6-76.1	73.2-75.3	73.9-74.8	75.4-76.3	74.5-75.8	87.2-97.7	100				
**CSSGNV**	64.7-65.0	63.5-63.7	73.5-76.0	72.0-75.4	71.3-72.1	72.8-73.8	73.3-74.4	77.4-79.2	78.9	100			
**CaMMV**	66.0-66.5	64.3-64.8	63.1-66.5	63.6-66.6	63.2-65.0	64.5-64.6	63.0-65.0	63.4-64.3	64.0	63.7	100		
**CYVBV**	63.7-63.8	64.2-63.9	63.3-65.6	63.4-64.6	65.6-66.4	64.9-65.4	62.9-63.9	63.9-64.9	63.7	66.1	62.1	100	
**CBSLV**	65.2-65.7	64.0-62.7	63.2-64.6	62.1-65.1	63.0-64.1	63.3-64.0	61.5-63.8	62.7-63.5	62.8	63.5	63.2	65	100
Complete genomes													
	**CSSGQV**	**CSSGRV***	**CRVV†**	**CSSCEV**	**CSSCDV**	**CSSTAV**	**CSSTBV**	**CRVBV⁑**	**CSSGMV**	**CSSGNV**	**CaMMV**	**CYVBV**	**CBSLV**
**CSSGQV**	99.3-99.6												
**CSSGRV***	72.8-73.6	98.6-99.8											
**CRVV†**	62.2-63.1	62.2-63.6	76.7-81.5										
**CSSCEV**	62.0-64.1	61.7-63.5	76.1-91.5	76.0-80.8									
**CSSCDV**	62.0-63.5	62.4-63.5	70.4-71.6	69.9-71.6	87.7-99.3								
**CSSTAV**	61.9-62.8	62.1-62.8	70.4-71.9	70.6-71.3	75.6-76.4	86.9							
**CSSTBV**	61.4-63.5	61.6-63.6	70.5-72.3	70.3-72.1	74.0-76.0	75.1-79.7	85.9-100						
**CRVBV⁑**	63.2-64.4	62.4-63.5	69.5-70.9	69.1-70.9	70.9-72.7	71.8-73.0	72.3-74.8	88.5-99.7					
**CSSGMV**	63.6-64.0	63.0-63.4	69.6-70.4	69.6-70.5	71.0-71.4	72.1-72.2	72.3-72.8	85.9-97.6	100				

**CSSGNV**	62.9-63.3	62.4-62.7	68.8-69.2	69.1-69.4	70.0-70.4	70.2-70.5	69.7-71.3	74.2-75.6	75.4	100			
**CaMMV**	60.5-61.0	61.1-61.9	60.6-62.2	61.3-61.7	60.6-61.4	60.7-61.6	60.6-61.8	61.2-62.1	61.7	61.4	100		
**CYVBV**	59.6-60.3	60.3-60.8	59.5-61.1	59.0-61.3	60.3-60.9	60.0-61.1	59.3-60.8	59.9-61.4	60.3	60.9	60.2	100	
**CBSLV**	61.0-61.2	59.6-59.8	60.8-61.3	59.7-61.5	60.6-61.1	60.6-60.7	59.2-60.8	59.5-61.2	60.1	60.4	60.4	60.8	100

* CSSGRV isolates belong to the accepted species CSSGQV

† CRVV isolates belong to the accepted species CSSCEV

⁑ CRVBV isolates belong to the accepted species CSSGMV

Supplementary Table S4. Predicted recombination events detected within badnavirus isolates infecting cacao, based on complete genome sequences.

Event	Breakpoints*		Recombinant	Parents		Methods†	*P* value‡
	Begin	End		Minor	Major		
1	168 (IR)	1023 (ORF2)	^L14546_CSSTBV<br>AJ534983_CSSTBV	AJ781003_CSSTAV	CSSTBV_Kipi7b	R**G**BMCS3	2.9E-57
2	27 (IR)	398 (ORF1)	^CSSTBV_Buyo17<br>CSSTBV_Buyo15<br>CSSTBV_Buyo7<br>CSSTBV_Buyo4<br>CSSTBV_Buyo5<br>CSSTBV_Kipi7b<br>CSSTBV_Kipi10b<br>CSSTBV_Kipi26	CSSCDV_Kipi7a	MF642717_CSSTBV	RG**B**MCS3	2.1E-23
3	637 (ORF1)	910 (ORF2)	CSSTBV_Buyo15<br>CSSTBV_Buyo17<br>CSSTBV_Buyo7<br>CSSTBV_Buyo4<br>CSSTBV_Buyo5<br>CSSTBV_Kipi7b<br>CSSTBV_Kipi10b<br>CSSTBV_Kipi26	MF642718_CSSCDV	KX592579_CSSTBV	**R**GBMCS3	4.8E-19
4	6959 (IR)	229 (IR)	^MF64273_CSSGQV	Unknown	MF642735_CSSGQV	**G**BMCS3	9.1E-18
5	7003 (IR)	91(?)	L14546_CSSTBV<br>AJ534983_CSSTBV	MF642716_CSSTAV	KX592574_CSSTBV	**R**GBMCS3	4.3E-16

6	4980 (ORF3)	6340 (ORF3)	^CSSTBV_Pbou21	MF642719_CSSCEV	AJ781003_CSSTAV	BMCS3	3.9E-07
			L14546_CSSTBV				
			AJ534983_CSSTBV				
			CSSTBV_Buyo15				
			CSSTBV_Buyo17				
			CSSTBV_Buyo7				
			CSSTBV_Buyo4				
			CSSTBV_Buyo5				
			CSSTBV_Kipi7b				
			CSSTBV_Kipi10b				
			CSSTBV_Kipi26				
			KX592574_CSSTBV				
			MF642717_CSSTBV				
			AJ608931_CSSTBV				
			KX592575_CSSTBV				
			AJ609019_CSSTBV				
			KX592578_CSSTBV				
			KX592577_CSSTBV				
			KX592579_CSSTBV				
			KX592580_CSSTBV				
			CSSTBV_Krag15				
			KX592582_CSSTBV				
			KX592583_CSSTBV				
			CSSTBV_Kipi12				
			KX592576_CSSTBV				
			KX592581_CSSTBV				
			CSSTBV_Krag1				
			CSSTBV_Krag5				
			CSSTBV_Pbou3				

			CSSTBV_Pbou12				
			CSSTBV_Krag8				
			CSSTBV_Krag9				
			CSSTBV_Krag10				
			CSSTBV_Pbou22				
			CSSTBV_Pbon23				
			CSSTBV_Pbon24				
			CSSTBV_Krag11				
			CSSTBV_Pbon2				
			CSSTBV_Pbon1				
			CSSTBV_Pbou1				
7	6841 (IR)	7030(?)	^KX592574_CSSTBV	Unknown	CSSTBV_Krag8	RGBMCS	3.8E-10
8	3925 (ORF3)	5053 (ORF3)	^KX592573_CSSCEV	MF642720_CSSCEV	KX592572_CSSCEV	RGBMCS3	3.1E-06

* Numbering starts at the 5′ end of the minus-strand primer-binding site and increases clockwise. (?), breakpoint could not be precisely pinpointed.

† R, RDP; G, GeneConv; B, Bootscan; M, MaxChi; C, Chimera; S, SisScan; 3, 3SEQ.

‡ The reported *P* values are for the methods indicated in red, and they are the lowest *P* values calculated for the region in question.

^ The recombinant sequence may have been misidentified (one of the identified parents might be the recombinant).

Supplementary Table S5. Predicted recombination events detected within badnavirus isolates infecting cacao, based on complete genomes available from GenBank, and Sanger sequences validated here (*Cacao swollen shoot Togo virus* [CSSTBV, isolate Buyo17] and *Cacao swollen shoot CD virus* [CSSCDV, isolate Buyo2]).

Event	Breakpoints*		Recombinant	Parents		Methods†	*P* value‡
	Begin	End		Minor	Major		
1	93(IR)	852(ORF2)	^CSSTBV_Buyo17	Unknown	L14546_CSSTBV	R**G**BMCS3	8.5E-58
2	168(IR)	816(ORF2)	^L14546_CSSTBV<br>AJ534983_CSSTBV	AJ781003_CSSTAV	KX592576_CSSTBV	R**G**BMCS3	5.2E-50
3	2265(ORF3)	3687(ORF3)	^MF642733_CSSGQV	MF642731_CSSGQV	Unknown	RGBMC**S**3	4.8E-16
4	1028(ORF2)	6990(IR)	^L14546_CSSTBV<br>AJ534983_CSSTBV	AJ609019_CSSTBV	MF642716_CSSTAV	RMC**S**3	1.4E-43
5	4473(ORF3)	6460(ORF3)	^CSSCDV_Buyo2<br>MF642718_CSSCDV_CIDivo<br>JN606110_CSSCDV_CI152	Unknown	MF642717_CSSTBV	BM**C**S3	5.6E-06
6	87(IR)	327(IR)	^MF642724_CSSGMV	Unknown	AJ609019_CSSTBV	**R**GBMCS	1.3E-04

* Numbering starts at the 5′ end of the minus-strand primer-binding site and increases clockwise.

† R, RDP; G, GeneConv; B, Bootscan; M, MaxChi; C, Chimera; S, SisScan; 3, 3SEQ.

‡ The reported *P* values are for the methods indicated in red, and they are the lowest *P* values calculated for the region in question.

^ The recombinant sequence may have been misidentified (one of the identified parents might be the recombinant).
